# FGF/FGFR-related lncRNAs based classification predicts prognosis and guides therapy in gastric cancer

**DOI:** 10.3389/fgene.2022.948102

**Published:** 2022-08-29

**Authors:** Qiuxiang Chen, Xiaojing Du

**Affiliations:** ^1^ Department of Ultrasound, The First Affiliated Hospital of Wenzhou Medical University, Wenzhou, Zhejiang, China; ^2^ Department of Gastroenterology, Minhang Hospital, Fudan University, Shanghai, China; ^3^ Key Laboratory of Diagnosis and Treatment of Severe Hepato-Pancreatic Diseases of Zhejiang Province, The First Affiliated Hospital of Wenzhou Medical University, Wenzhou, China

**Keywords:** gastric cancer, FGF/FGFR, lncRNAs, immune infiltration, precision medicine

## Abstract

Fibroblast growth factor (FGF) and its receptor (FGFR) play crucial roles in gastric cancer (GC). Long non-coding RNAs (lncRNAs) are defined as RNA molecules of around 200 nucleotides or more, which are not translated into proteins. As well-known regulatory factors, lncRNAs are considered as biomarkers for prognosis and treatment response in GC. It is of importance to identify FGF/FGFR-related lncRNAs in GC. Here, some FGF/FGFR-related lncRNAs were identified in GC based on the data from public databases, the Cancer Genome Atlas (TCGA) and Gene Expression Omnibus (GEO). Then a four-lncRNAs (*FGF10-AS1*, *MIR2052HG*, *POU6F2-AS2*, and *DIRC1*) risk score (RS) model was established for predicting GC’s prognosis by using Cox analysis. According to the median value of RS, GC patients were divided into low and high RS group. Low RS group displayed high tumor mutation burden and infiltration of immune cells, as well as more sensitivity to immunotherapy or chemotherapy. High RS group showed high infiltration of stromal cells and more oncogenic signatures. In addition, a comprehensive analysis was carried out and found that high RS group may exhibit specific sensitivity to Panobinostat (histone deacetylases inhibitor) and Tivantinib (MET inhibitor). In summary, our study not only offers a novel personalized prognostication classification model according to FGF/FGFR-related lncRNAs, but also provides a new strategy for subclass-specific precision treatment in GC.

## Introduction

The development of precision medicine has greatly changed the outlook of many patients with advanced gastric cancer (GC) in recent decades. Unfortunately, high genomic heterogeneity becomes the major barrier to precision medicine in GC (Pectasides et al., 2018). GC remains the third leading cause of cancer-related death worldwide (Siegel et al., 2021). Therefore, improvement of our understanding to the molecular characteristic underlying GC development is of great importance to its therapy and patients’ prognosis.

Fibroblast growth factor (FGF) and its receptor (FGFR) belong to the tyrosine kinase receptor (RTK) family, which include 22 ligands (FGF1-FGF14, FGF16-FGF23) and 5 receptors (FGFR1-4 and FGFRL1) (Zhou et al., 2020). Several pre-clinical and clinical evidences have indicated that some FGF/FGFR family members, such as FGFR1, FGFR2, FGFR3, FGFR4, FGF7, FGF9, FGF10, and others, played crucial roles in GC’s progression (Zhang et al., 2019). Consequently, identifying the regulatory mechanism of FGF/FGFR may conduce to understanding the molecular mechanism underlying GC.

Long non-coding RNAs (lncRNAs) are defined as a group of RNA molecules that can’t translate to proteins (Palazzo and Koonin, 2018). LncRNAs are longer than 200 nucleotides and could act in cis or in trans for exerting enhancer or silencer regulation on gene expression (Palazzo and Koonin, 2018). A growing body of studies revealed that lncRNAs could serve as biomarkers for diagnosis, prognosis, and responses to treatment of GC (Yuan et al., 2020). Here, a series of FGF/FGFR-related lncRNAs with prognosis signature were identified in GC. Then, these lncRNAs were used to construct a risk score (RS) model and divide GC samples into two groups (low RS group and high RS group). The clinical, molecular and tumor microenviroment (TME) characteristics were further investigated for the two groups. Finally, a subclass-specific therapy strategy was also identified.

## Materials and methods

**FIGURE 1:**
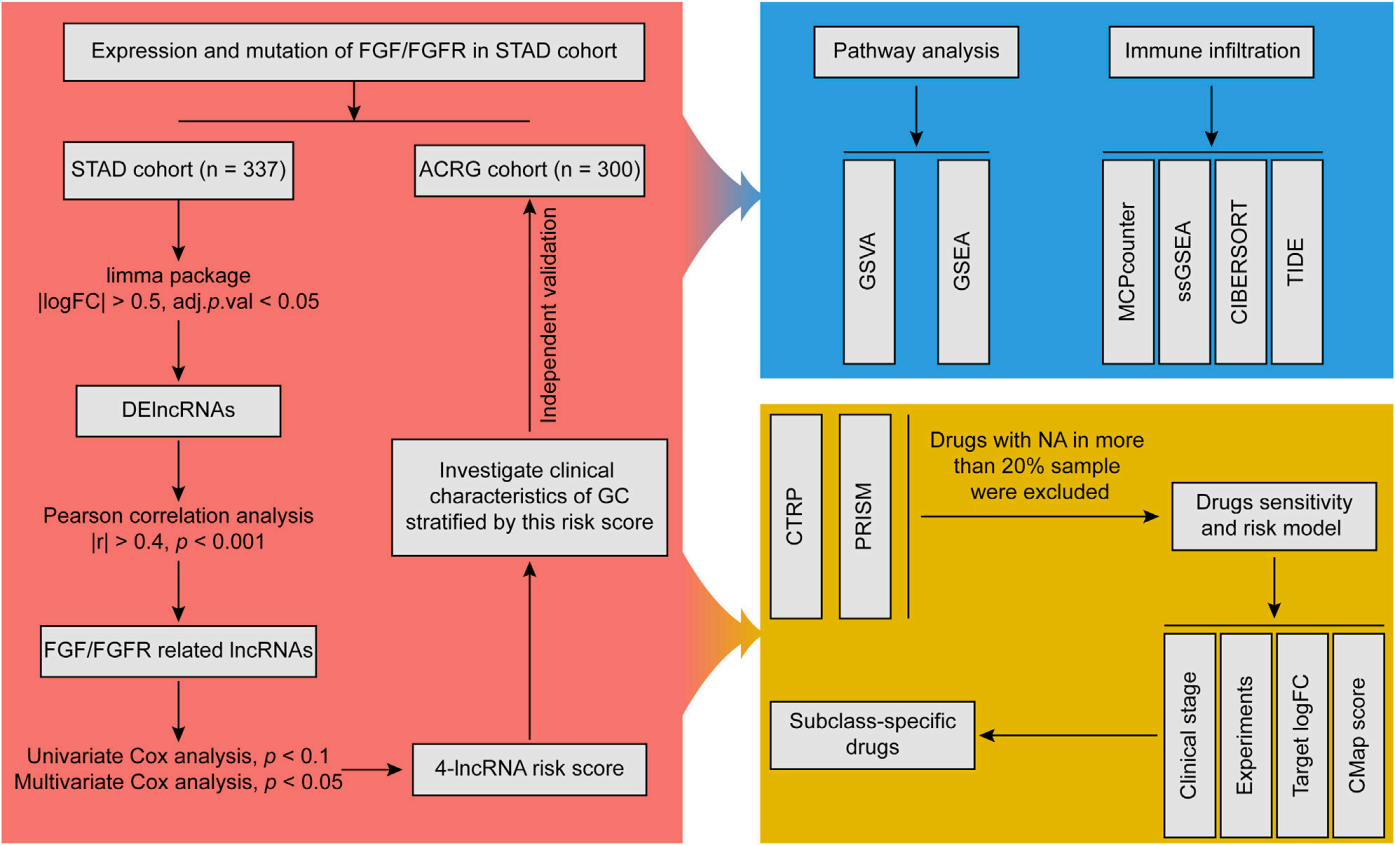
Analytic flowchart.

The overall analysis processes were shown in Figure 1.

### Data sources and processes

RNA-sequencing cohort stomach adenocarcinoma (STAD, 32 normal and 375 tumor samples) was downloaded from the Cancer Genome Atlas (TCGA) database (Cancer Genome Atlas Research Network, 2014). The mRNAs and lncRNAs were annotated with annotation file from GENCODE database (Frankish et al., 2021). The pathology image data were also downloaded from TCGA database. Microarray cohort GSE62254 (300 tumor samples, Asian Cancer Research Group, ACRG cohort) was obtained from Gene Expression Omnibus (GEO) database and annotated with GPL570 platform (Cristescu et al., 2015). The clinical characteristics of these GC samples are shown in Supplementary Table S1. Drug sensitivity data of human cancer cell lines (CCLs) were downloaded from Cancer Therapeutics Response Portal (CTRPv.2.0, released October 2015) and PRISM Repurposing dataset (19Q4, released December 2019) (Rees et al., 2016; Corsello et al., 2020). The area under the dose-response curve (AUC) values were used to estimate drug sensitivity (AUC values in CTRP v.2.0 range from 0 to 30, in PRISM range from 0 to 1). Before processing, drugs that were not available (NA) in more than 20% of the samples were excluded, and the remaining NA was imputed by the k-nearest neighbors (KNN) algorithm. The gene expression data of CCLs were obtained from Cancer Cell Line Encyclopedia (CCLE) project (Ghandi et al., 2019).

### Differential gene analysis

The limma package of R (version 4.1.0) was used to analyze the differential expression of FGF/FGFR family between normal and tumor tissues in STAD cohort (Ritchie et al., 2015). In addition, the differential expressed lncRNAs (DElncRNAs) were also calculated by limma package, setting the threshold as |log_2_ (fold change) (logFC)| > 0.5 and adjust *p* value (adj.*p*.val) < 0.05.

### Somatic mutation analysis

Tumor mutational data (maf file) of GC were downloaded from TCGA database, and the mutations of FGF/FGFR family and other pathway-related genes were analyzed by the “maftools” package of R (Mayakonda et al., 2018). Tumor mutation burden (TMB) of GC was calculated by using “TCGAmutations” package of R (Ellrott et al., 2018).

### Construction and validation of long non-coding RNA-based risk score

To construct a survival-related RS model, STAD cohort was used as the training cohort and GC samples who have complete follow-up information and follow up time over 30 days were included. At first, FGF/FGFR-related lncRNAs were identified by using Pearson correlation analysis with the cutoff values as followed: |r| > 0.4 and *p* < 0.001. Then, lncRNAs with *p* < 0.1 measured by univariate Cox analysis were selected for a further multivariate Cox analysis in STAD cohort, and lncRNAs with *p* < 0.05 were considered as the hub lncRNAs. The calculation formula for RS was showed as followed:

$$\text{RS}=\sum {\text{Coef}}_{\text{lncRNAs}}{\text{ × Exp}}_{\text{lncRNAs}}$$

Coef_lncRNAs_ means the regression coefficient of lncRNAs calculated by multivariate Cox analysis. For the RNA-sequencing data, Exp_lncRNAs_ represents log_2_ (count +1) value of the involved lncRNA. For the micrroarray data, Exp_lncRNAs_ indicates the raw value of the involved lncRNA that was provided by GEO database. According to the median value, patients were divided into low and high RS groups. Kaplan-Meier survival analysis was achieved by the “survival” package. Decision curve analysis (DCA) was employed to test the efficacy of RS in both training cohort (STAD) and validation cohort (ACRG) (Vickers and Elkin, 2006).

### Gene set variation analysis

GSVA was applied to enrich oncogenic signature (c6.all.v7.5.1. symbols) from Molecular Signatures Database (MSigDB) using the clusterProfiler package (Subramanian et al., 2005; Wu et al., 2021). The package of limma was employed to analyze the differential pathways between low and high RS group.

### Gene set enrichment analysis

The fold change of each gene between low and high RS group was firstly calculated by limma package, and genes were input in descending order according to the logFC values. GSEA was used to enrich gene set “c6.all.v7.5.1. symbols” using the clusterProfiler package (Subramanian et al., 2005; Wu et al., 2021). An adj. *p*.val less than 0.05 was considered as a significantly enrichment.

### Tumor microenviroment analysis

Estimate algorithm was used to calculate stromal, immune, estimate score and tumor purity (Yoshihara et al., 2013). MCPcounter, ssGSEA and CIBERSORT algorithm were applied to measure the level of infiltration cells (Newman et al., 2015; Becht et al., 2016; Chen et al., 2018). Cancer immunity cycle included seven steps, and related gene sets were acquired from the tracking tumor immune phenotype (TIP) website and quantified using the ssGSEA algorithm (Xu et al., 2018). The response to immunotherapy was predicted by Tumor Immune Dysfunction and Exclusion (TIDE) algorithm (Jiang et al., 2018).

### Connectivity map analysis

A standardized score ranging from −100 to 100 can be calculated by CMap analysis for each perturbation. A negative score means that an expression pattern of perturbation is opposite to the disease expression pattern, prompting a potential therapeutic value of this perturbation in this disease. Accordingly, we firstly conducted differential analysis between tumor and normal samples. Then, the top 150 upregulated and downregulated genes were submitted to the CMap website, and the CMap score was calculated (Subramanian et al., 2017).

### Estimating drug sensitivity in stomach adenocarcinoma cohort

The “pRRophetic” package of R was used to estimate the sensitivity to chemotherapeutic drugs (Cisplatin, Docetaxel, Doxorubicin, Etoposide, and Paclitaxel) and targeted drugs (Pazopanib, Sunitinib, Gefitinib, and Erlotinib) in STAD cohort (Geeleher et al., 2014).

### Statistical analysis

R software (v4.1.0, R Core Team, R Foundation for Statistical Computing, Vienna, Austria) was employed for statistical analysis. The student’s *t*-test or Mann Whitney test was used to compare the difference between two RS groups. Correlation analysis was achieved by Pearson correlation analysis. Survival analysis was conducted by using Kaplan-Meier methods and the statistical significance of differences was determined by a log-rank test. If not specified above, a two-tailed *p* < 0.05 was considered statistically significant.

## Results

### Overall landscape of fibroblast growth factor and its fibroblast growth factor receptor in gastric cancer

**FIGURE 2:**
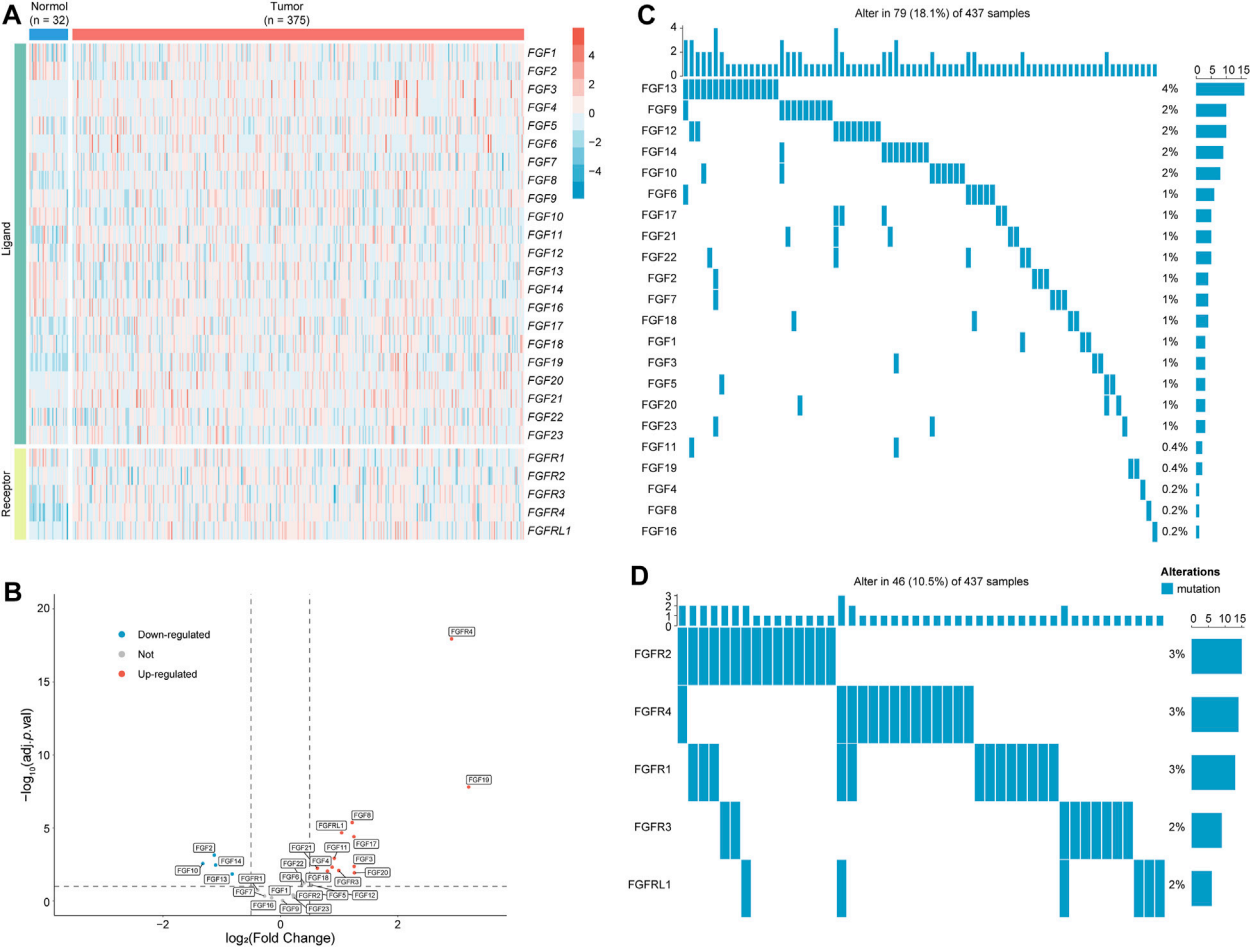
The expression and mutation of FGF/FGFR in GC. **(A,B)** The expression of FGF/FGFR family members in normal and GC tumor tissues was shown in heatmap **(A)** and volcano plot **(B)**. **(C)** The mutation rate of FGFs in GC. **(D)** The mutation rate of FGFRs in GC. FGF, fibroblast growth factor; FGFR, fibroblast growth factor receptor; GC, gastric cancer.

FGF/FGFR family is comprised of 22 ligands and 5 receptors. Previous studies demonstrated the crucial roles of FGF/FGFR played in GC (Zhang et al., 2019). To evaluate the overall landscape of FGF/FGFR in GC, the expression level and mutation rate of FGF/FGFR were firstly analyzed. Results showed that *FGF3*, *FGF4*, *FGF8, FGF17*, *FGF18*, *FGF19*, *FGF20*, *FGF21*, *FGFR3*, *FGFR4*, and *FGFRL1* were upregulated in GC, while *FGF2*, *FGF10*, *FGF13*, and *FGF14* were downregulated (Figures 2A,B). Somatic mutation analysis indicated that *FGF13* had the highest mutation rate of 4% in all ligands (Figure 2C). Among the receptors, *FGFR1*, *FGFR2*, and *FGFR4* shared the highest mutation rate of 3% (Figure 2D). Next, the correlation between these genes and GC patients’ survival was analyzed *via* Kaplan-Meier Plotter online tool (Szász et al., 2016). Besides *FGF20* and *FGF22*, other FGF/FGFR family members were significantly negative correlation with GC patients’ prognosis (Supplementary Figure S1). Accordingly, we and others indicated that FGF/FGFR family played crucial roles in GC (Zhang et al., 2019; Zhou et al., 2020).

### Identification of fibroblast growth factor and its receptor related long non-coding RNAs and construction of a prognostic risk score model

LncRNAs serve as a major modulation of gene expression. To seek the potential mechanisms underlying the aberrant FGF/FGFR in GC, FGF/FGFR related lncRNAs were identified *via* the following processes. At first, 5,598 DElncRNAs (408 downregulated and 5,190 upregulated lncRNAs) were identified between normal and tumor tissues in STAD cohort, among which 657 lncRNAs were also detected in ACRG cohort (Supplementary Figures S2A,B). The correlation between the FGF/FGFR family and these overlapped lncRNAs were analyzed by using Pearson correlation analysis (Supplementary Table S2). A total of 235 lncRNAs (|r| > 0.4, *p* < 0.001) were selected as FGF/FGFR-related lncRNAs in GC. Of note, all of these lncRNAs were positive correlation with the expression of FGF or FGFR. In addition, the location of these genes in chromosome was visualized by using TBtools (Chen C et al., 2020). The results showed that some members of FGF/FGFR family are in the neighbourhood of these lncRNAs, such as *FGF10-AS1* and *FGF10*, *LINC01537* and *FGF3* or *FGF4*, *LBX-AS1* and *FGF8*, *MIR497HG* and *FGF11*, *etc.* (Supplementary Figure S3). Taken together, these lncRNAs may act potential regulation effects on the expression of FGF/FGFR, and further investigations may help us in understanding the mechanisms of aberrant FGF/FGFR in GC.

**FIGURE 3:**
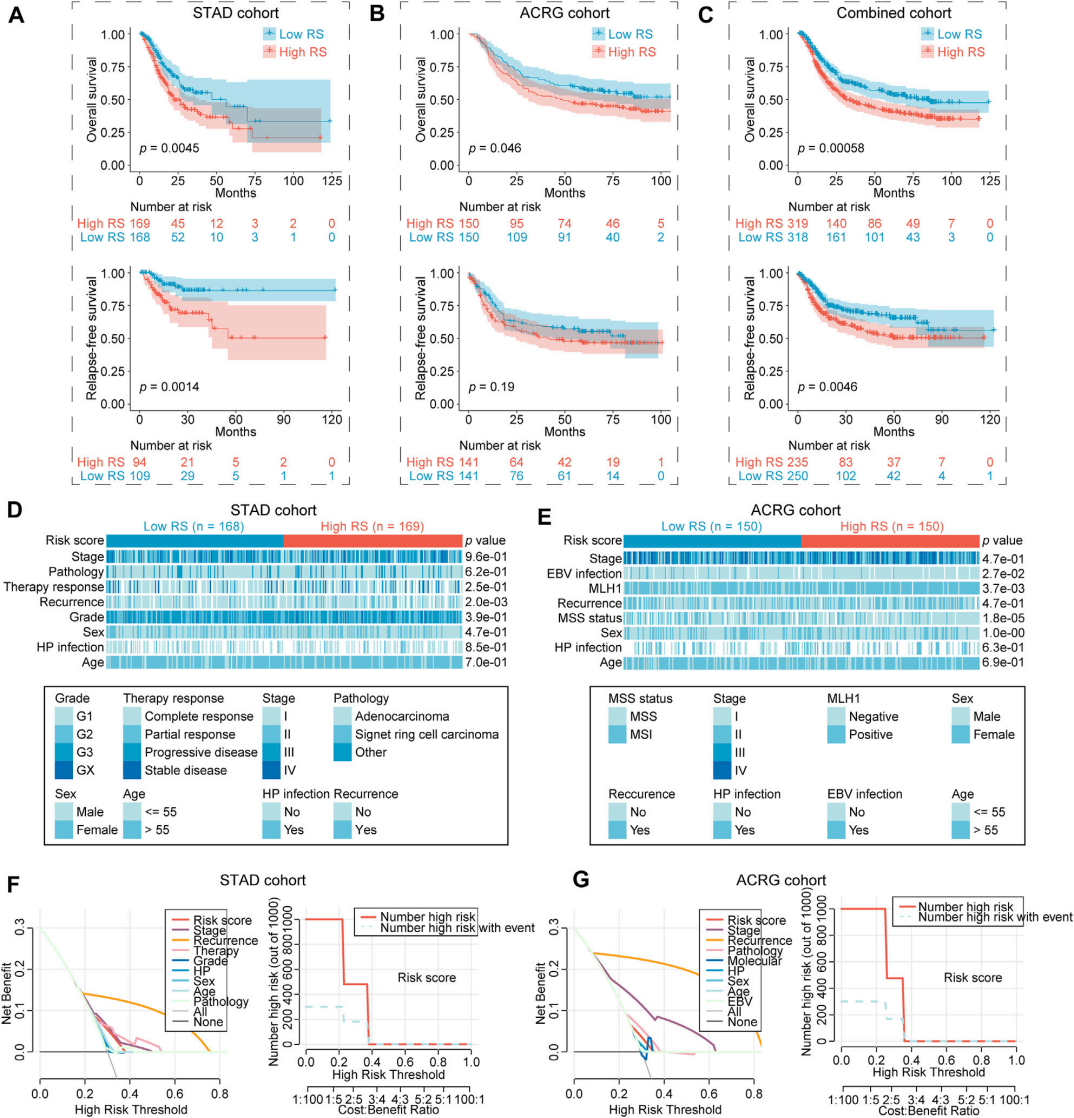
Significant difference of clinical characteristics in the two RS groups. **(A–C)** Overall survival and relapse-free survival of low and high RS group in STAD cohort **(A)**, ACRG cohort **(B)**, combined cohort (STAD + ACRG) **(C)**. **(D,E)** Clinical characteristics of low and high RS group in STAD **(D)** and ACRG cohort **(E)**. Statistical significance of difference was determined using Chi-Square test or Fisher’s exact tests. **(F,G)** DCA of clinical characteristics in STAD **(F)** and ACRG cohort **(G)**. RS, risk score; STAD, stomach adenocarcinoma; ACRG, Asian Cancer Research Group; MSS, microsatellite stability; MSI, microsatellite instability; DCA, decision curve analysis.

LncRNAs were also considered as a potential prognosis biomarker for GC (Yuan et al., 2020). Hence, a RS model for predicting patients’ prognosis was established by using Cox analysis. Firstly, univariate Cox analysis was employed to screen lncRNAs with prognostic signature in GC, and 45 lncRNAs (*p* < 0.1) were selected for a multivariate Cox analysis (Supplementary Table S3). Then, *FGF10-AS1*, *MIR2052HG*, *POU6F2-AS2*, and *DIRC1* were identified as the hub lncRNAs and a RS formula was established as followed: RS = 0.78 × Exp_*FGF10-AS1*_ + 0.87 × Exp_*MIR2052HG*_ + 1.12 × Exp_*POU6F2-AS2*_ + 1.15 × Exp_*DIRC1*_ (Supplementary Table S4). Subsequently, survival analysis was used to investigate the correlation between RS and GC’s prognosis. The results indicated a significant prognostic difference between the low and high RS group in STAD cohort when using overall survival (OS) as an endpoint, the low RS group (*n* = 168) had a longer median OS time (mOS) than the high RS group (*n* = 169, 56.2 vs. 22.5 months, *p* = 0.0045; Figure 3A). Consistent results were observed in the validation ACRG cohort, and the high RS group (*n* = 150, mOS = 48.2 months) exhibited a shorter mOS than the low RS group (*n* = 150, mOS not reached, *p* = 0.046) (Figure 3B). Patients’ OS was gradually decreased along with the increasing RS, and the expression of each hub lncRNA was shown in the heatmap (Supplementary Figure S4). Setting relapse-free survival (RFS) as an endpoint, a significant difference was also observed between the low and high RS group in STAD cohort (*p* = 0.0014). Although there was no significant RFS difference between the two groups in ACRG cohort, it was still obvious that the low RS group was associated with the tendency toward longer RFS time (median RFS time mPFS 81.4 vs. 40.8 months, *p* = 0.19). Then, the respective subclass and clinical information of cases in training and validation cohorts were combined together. Based on the combined data, significant difference between two RS groups was also observed when comparing OS (*p* = 0.00058) or RFS (*p* = 0.0046; Figure 3C). These data suggested that this RS model was significant association with GC’s prognosis.

### Clinical characteristics of gastric cancer in different risk score groups

At first, the association between RS and clinical characteristics was analyzed in GC. RS was correlated with high recurrence rate (*p* < 0.01), high MLH1 positive (*p* < 0.01), more microsatellite stability (MSS) (*p* < 0.0001), as well as less EBV infection (*p* < 0.05; Figures 3D,E). We further evaluated that wheather recurrence rate, MLH1 level, microsatellite status and EBV infection performed a prognosis significance in GC. The results showed that except EBV (*p* = 0.87), recurrence rate (*p* < 0.0001), MLH1 level (*p* = 0.0022) as well microsatellite status (*p* = 0.016) were also significantly with GC’s OS. Patients with recurrence (18.5 months vs. not reached), MLH1 positive (50.3 months vs. not reached) and/or MSS (65.0 months vs. not reached) had a shorter OS (Supplementary Figure S5). Decision curve analysis (DCA) is a potent tool for estimating the efficacy of clinical model (Vickers and Elkin, 2006). Hence, DCA was employed to compare the prediction efficacy of this RS model and other clinical parameters in GC. The results showed that RS performed well prediction efficacy in both STAD and ACRG cohort, but unfortunately it was still inferior to several clinical characteristics, such as stage, recurrence, and therapy response (Figures 3F,G).

### Molecular characteristics of gastric cancer in different risk score groups

**FIGURE 4:**
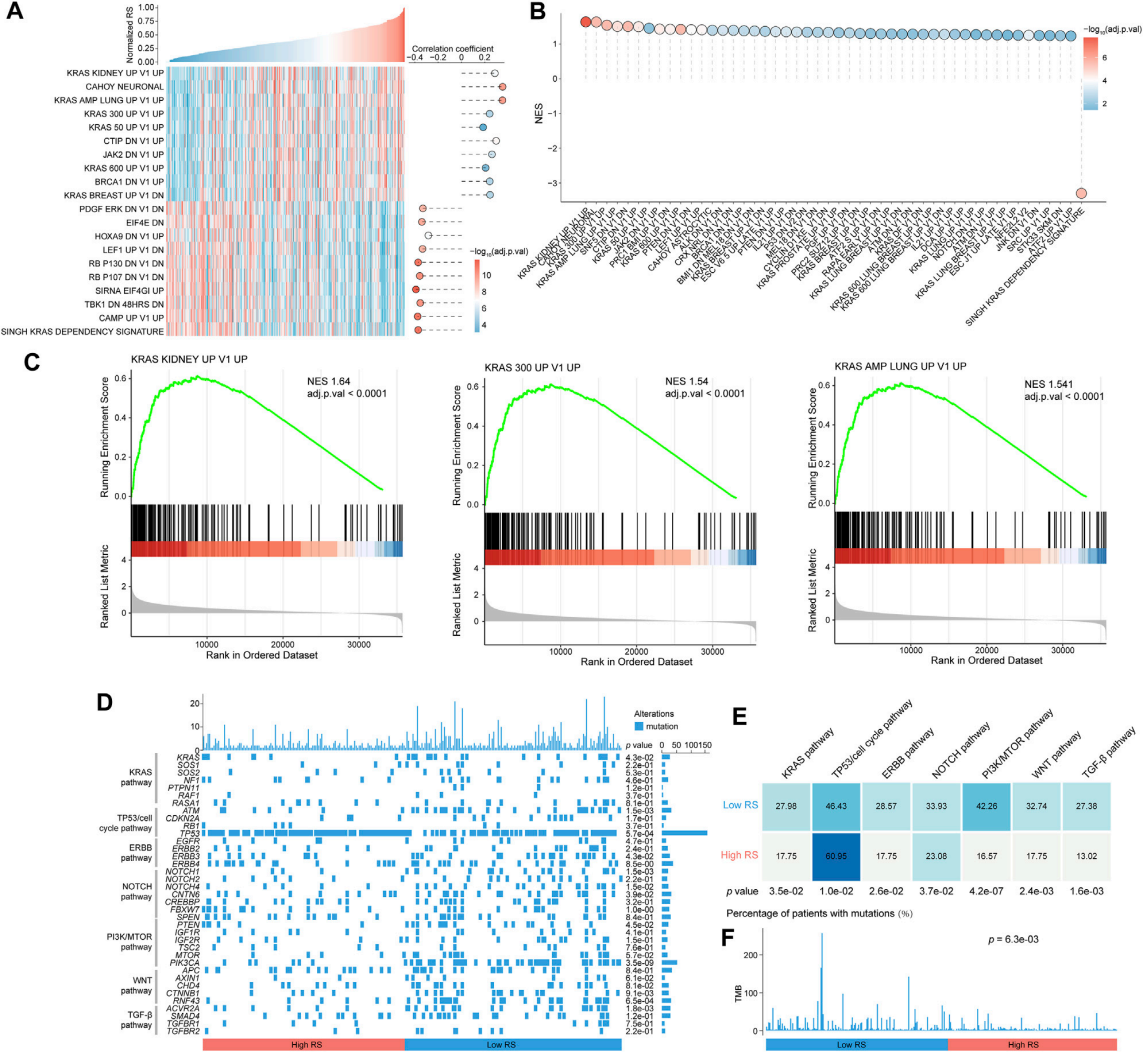
Significant difference of molecular characteristics in the two RS groups. **(A)** Overview of the association between RS and oncogenic signatures scored by GSVA. The correlation coefficient and significance are measured by using Pearson correlation analysis. **(B)** The result of GSEA shows high RS-associated and low RS-associated oncogenic signatures. **(C)** Three representative GSEA enrichment plots. **(D)** Oncoprint of mutation status of genes in several key pathways engaged in GC. Statistical significance of difference was analyzed using Chi-Square test or Fisher’s exact test. **(E)** Percentage of mutations in different pathways between two RS groups. **(F)** TMB in the two RS groups. Chi-Square test was used to achieve statistical analysis. RS, risk score; GSVA, Q14 gene set variation analysis; GSEA, gene set enrichment analysis.

Next, the molecular characteristics was revealed between the two RS groups in STAD cohort. GSVA was firstly performed using expression profiles from tumor samples to calculate gene set enrichment score. The high RS group was mainly associated with KRAS-related oncogenic signature (Figure 4A; Supplementary Table S5). GSEA of oncogenic signature suggested that the upregulated genes in the high RS group were enriched in KRAS, PTEN, TP53, and JAK2 related gene sets (Figures 4B,C; Supplementary Table S6). Of note, most of FGF/FGFR family members were upregulated in the high RS group compared to the low RS group, while no significant difference was observed in the expression of *KRAS*, *HRAS*, and *NRAS* between two RS groups (Supplementary Figure S6). These data demonstrated that the high RS group possessed more deregulated oncogenic signatures, which may be due to the aberrant FGF/FGFR in GC.

Gene mutations are also the important driving factors in GC. Hence, we focused on the mutation status of genes involved in major pathways in GC, including KRAS pathway, TP53/cell cycle pathway, ERBB pathway, NOTCH pathway, PI3K/mTOR pathway, WNT pathway and TGF-β pathway, as shown in the waterfall plot (Figure 4D). Statistical results uncovered that patients in the high RS group had more frequent mutations in TP53/cell cycle pathway than those in the low RS group (60.95% vs. 46.43%, *p* < 0.05). The low RS group showed a higher percentage of mutations in KRAS pathway (27.98% vs. 17.75%, *p* < 0.05), ERBB pathway (28.57% vs. 17.75%, *p* < 0.05), NOTCH pathway (33.93% vs. 23.08%, *p* < 0.05), PI3K/mTOR pathway (42.26% vs. 16.57%, *p* < 0.0001), WNT pathway (32.74% vs. 17.75%, *p* < 0.01) and TGF-β pathway (27.38% vs. 13.02%, *p* < 0.01) compared to the high RS group (Figure 4E). The total TMB was also assessed between two risk groups, and results showed that the low RS group had a higher TMB than the high RS group (*p* < 0.01; Figure 4F).

### The differences of tumor microenviroment in the two risk score groups

**FIGURE 5:**
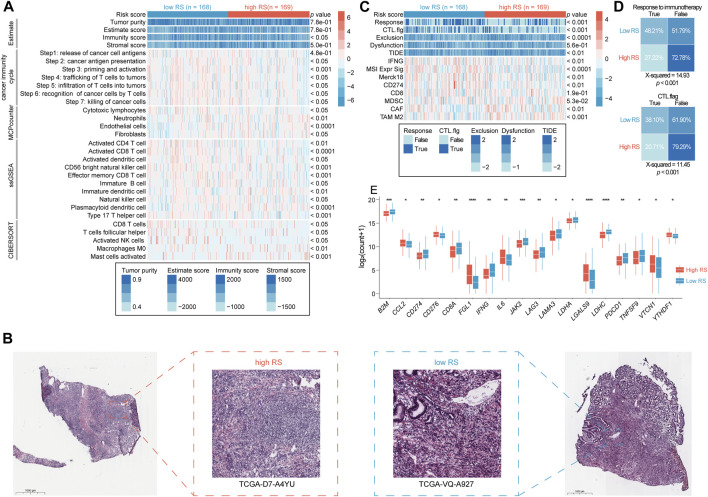
Different immune microenvironment in the two RS groups. **(A)** The heatmap shows the frequency of tumor microenvironment infiltrating cells and immune score between the two RS groups (student’s *t*-test or Mann Whitney test). **(B)** The representative slide images of tumor in the two RS groups. **(C)** The heatmap shows TIDE score between the two RS groups (student’s *t*-test or Mann Whitney test). **(D)** The response rate to immunotherapy between the two RS groups (Chi-Square test). **(E)** The mRNA expression of immune checkpoint genes in the two RS groups. *p < 0.05, **p < 0.01, ***p < 0.001, and ****p < 0.0001. RS, risk score; TIDE, Tumor Immune Dysfunction and Exclusion.

Moreover, the TME characteristics were investigated between the two RS groups. To this end, the TME score was determined by using estimate algorithm. The results showed that the low RS group had a higher immune score than the high RS group (576.4 vs. 362.0, *p* < 0.05; Figure 5A). Immune cells and stromal cells are the major constituent cells in TME. The infiltration of immune cells and stromal cells was next analyzed in GC. The results showed that the low RS group had higher abundance of immune cells, including activated CD4 T-cells (*p* < 0.01), activated CD8 T-cells (*p* < 0.0001), activated dendritic cells (DCs, *p* < 0.05), CD56 bright natural killer cells (NK cells, *p* < 0.001), effector memory CD8 T-cells (*p* < 0.001), immature B cell (*p* < 0.05), NK cells (*p* < 0.05), type 17 T help cells (Th17 cells, *p* < 0.001), than high RS group (Figure 5A). CIBERSORT algorithm also showed higher infiltration of immune cells (e.g., CD8 T-cells, activated NK cells, T-cells follicular helper) in the low RS group rather than in high RS group. MCP counter algorithm indicated that the high RS group had higher infiltration of stromal cells, including fibroblasts (*p* < 0.05) and endothelial cells (*p* < 0.0001), compared to the low RS group (Figure 5A). Pathology images showed that more lymphocytes infiltrated into tumor in low RS samples (Figure 5B). TIDE algorithm was further used to estimate T-cell function and exclusion score (Jiang et al., 2018). Although results showed no significant differences of dysfunction score between two groups, the high RS group had a higher exclusion score than the low RS group (*p* < 0.0001), accounting for the lower T-cell infiltration in the former (Figures 5C,D). Taken together, the TME of low RS tumor was characterized by enriched immune cells, while high RS tumor by more stromal cells.

Cancer immunity cycle is considered as the most important pathway for immune cells’ works, which included seven steps: 1) Release of cancer cell antigens, 2) cancer antigen presentation, 3) priming and activation, 4) trafficking of T-cells to tumors, 5) infiltration of T-cells into tumors, 6) recognition of cancer cells by T-cells, and 7) killing of cancer cells (Xu et al., 2018). The score of these seven steps was calculated by ssGSEA algorithm. The results showed that these pathways were active in the low RS group, especially step 2–7 (Figure 5A), indicating that these GC patients may have a more active immune microenvironment. TIDE algorithm could be used to predict pateints’ response to immunotherapy. The results showed that the low RS group had a higher response rate to immunotherapy than the high RS group did (48.21% vs. 27.33%, *p* < 0.001; Figure 5D). Then, we analyzed the expression of immune checkpoint genes in tumor samples. Results suggested that the low RS group had a higher expression of eleven immune checkpoint genes, including *CD274* (PD-L1) and *PDCD1* (PD-1) than the high RS group (Figure 5E). In addition, the low RS group possessed a higher level of TMB (Figure 4F). These observations may explain the better response to immunotherapy of the low RS group.

### Identifying subclass-specific treatment strategies for gastric cancer

As abovementioned, two RS groups have different clinic, molecule and TME characteristics, indicating that this model had a preferable ability of classification for GC. Hence, we hypothesized that this two groups may have different response to distinct treatment. To prove this, we estimated the sensitivity to several clinical drugs between the two RS groups. Among chemotherapeutic drugs, the low RS cohort displayed more sensitivity to Etoposide (*p* < 0.01) and Paclitaxel (*p* < 0.01) than the high RS cohort did, and no significant difference was observed in the estimation of Cisplatin (*p* > 0.05), Docetaxel (*p* > 0.05) as well as Doxorubicin (*p* > 0.05) (Supplementary Figure S7). Among targeted drugs, the high RS cohort exhibited more sensitivity to Pazopanib (*p* < 0.0001) compared to the low RS cohort, but no significant difference in Sunitinib (*p* > 0.05), Gefitinib (*p* > 0.05) as well as Erlotinib (*p* > 0.05) (Supplementary Figure S7). In addition, above data indicated that GC patients in the low RS group had a higher response to immunotherapy than those in the high RS group (Figure 5D). Accordingly, immunotherapy or chemotherapy may be suitable for the low RS cohort.

**FIGURE 6:**
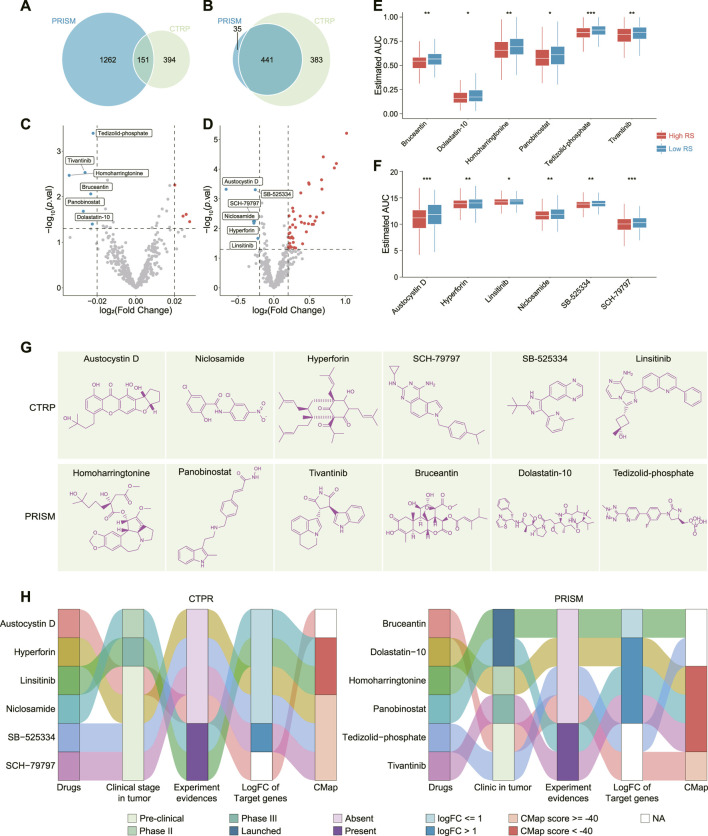
Comprehensive analysis of these candidate drugs. **(A,B)** Venn plot showed the drugs **(A)** and cell lines **(B)** included in PRISM and CTRP database. **(C,D)** Volcano plot displayed the drugs’ differential AUC values between low and high RS groups in PRISM **(C)** and CTRP **(D)** database. **(E)** AUC values of Homoharringtonine, Panobinostat, Tivantinib, Bruceantin, Dolastatin-10, and Tedizolid-phosphate between low and high RS groups. **(F)** AUC values of Austocystin D, Niclosamide, Hyperforin, SCH-79797, SB-525334, and Linsitinib between low and high RS groups. **(G)** Chemical structures of the twelve candidate drugs. **(H)** A comprehensive analysis of the twelve candidate drugs from clinical and experimental evidences, fold change of drug targeted genes, as well as CMap score. *p < 0.05, **p < 0.01, ***p < 0.001. RS, riskscore; CTRP, Cancer Therapeutics Response Portal; AUC, area under the dose-response curve; GC, gastric cancer; CMap, connectivity map; logFC, log_2_ (fold change).

To identify subclass-specific agents for the high RS cohort, the AUC values that were used to estimate drug sensitivity (lower AUC means more sensitivity) were downloaded from two large-scale datasets (CTRP and PRISM). In particular, the data of 545 drugs and 824 CCLs were downloaded from CTRP, and 1,419 drugs and 476 CCLs from PRISM (Supplementary Tables S7,8). CTRP and PRISM dataset shared 151 drugs and 85 CCLs (Figures 6A,B). Two exclusion criteria were used to select the data from these two databases: Drugs with NA in more than 20% of the samples were excluded; CCLs that have no expression data in CCLE dataset were excluded. After precondition, 405 drugs and 824 CCLs in CTRP, and 433 drugs and 476 CCLs in PRISM were retained. Subsequently, using RS scored CCLs and divided them into low and high RS group. Differential analysis of AUC values between two groups was carried out. According to the cutoff values for CTRP (logFC < -0.2, *p* < 0.05), six drugs, including Austocystin D (natural product, inducer of DNA damage), Niclosamide (STAT3 inhibitor), Hyperforin (TRPC6 inhibitor), SCH-79797 (F2R inhibitor), SB-525334 (TGFBR1 inhibitor) and Linsitinib (IGF1R inhibitor) were focused on; for PRISM (logFC < −0.02, *p* < 0.05), another six drugs, including Homoharringtonine (RPL3 inhibitor), Panobinostat (Histone deacetylases HDACs inhibitor), Tivantinib (MET inhibitor), Bruceantin (protein synthesis inhibitor), Dolastatin-10 (TUBB inhibitor) and Tedizolid-phosphate (protein synthesis inhibitor) were selected (Figures 6C–F). The chemical structure of these drugs were displayed (Figure 6G). These candidate drugs may exhibit outstanding effects against the high RS group. To further identify potential drugs, a multiple-perspective investigation was applied to comprehensively evaluate the therapeutic potential of these twelve candidate compounds in GC (Figure 6H). First, the clinical evidence of these compounds in treating tumor was collected from FDA approved drug list and ClinicalTrials website. Second, a comprehensive literature retrieval was performed in PubMed to search the experimental evidence of these compounds in treating GC. Third, logFC of the mRNA expression of drugs’ targets between the high RS tumor and normal tissue were calculated, and logFC >1.0 indicated a greater potential target. Forth, CMap analysis was employed to confirm the drugs that yield gene expression patterns oppositional to the GC expression patterns. Accordingly, two drugs, Panobinostat and Tivantinib were identified as the potential drugs for treating the high RS cohort.

## Discussion

In this work, a set of FGF/FGFR-related lncRNAs were identified. Based on Cox analysis, a four-lncRNA RS model was established for clustering GC and predicting GC’s prognosis. The low RS group performed a longer OS and RFS, higher mutation rate and immune infiltration, while the high RS group possessed enriched oncogenic signatures and more stromal cell infiltration. We also put forward subclass-specific treatment strategy for GC: Chemotherapy and immunotherapy may be more suitable for the low RS group, while targeted therapy (Panobinostat and Tivantinib) for the high RS group.

Majority of studies confirm that the FGF/FGFR family have achieved driving effects in the progression of GC (Zhang et al., 2019; Zhou et al., 2020). We observed that compared to normal tissues, some FGF/FGFR (e.g., *FGFR3*, *FGFR4*, *FGF3*, and *FGF4*) were up-regulated in GC tissues, while some (e.g., *FGF2* and *FGF10*) were downregulated in GC tissues. However, almost all of the members in FGF/FGFR family (besides *FGF20* and *FGF22*) were negative association with GC’s prognosis. For the upregulated members, it is easy to understand their association with prognosis in GC. For the downregulated or no significantly changed members, although the overall level of FGF/FGFR was lower than normal tissues, some tumor tissues still expressed high level of FGF or FGFR. In the cases with high FGF/FGFR expression, FGF/FGFR may also exert driving function for cancer progression, resulting in a worse prognosis than those with low FGF/FGFR expression. Besides amplification, mutation was also the common mechanism for FGF/FGFR’s cancer promoting effect (Katoh, 2019). Our study has also confirmed the occurrence of FGF/FGFR mutations in GC, being consistent with previous studies (Zhang et al., 2019), which was considered as a supplementary mechanism for their driving role in GC.

Solid evidences confirmed the regulatory function of lncRNAs in the gene expression (Statello et al., 2021). Several studies demonstrated the regulatory function of lncRNAs (*NEAT1*, *GAS5*, *NORAD*, and *Linc00460*) on some FGF/FGFR (*FGF1*, *FGF2*, *FGF7*, and *FGF21*) (Wang et al., 2019; Zhu et al., 2019; Chen H et al., 2020; Zhao et al., 2020), indicating that this regulatory mode also takes effect on the expression of FGF/FGFR. Here, a group of FGF/FGFR-related lncRNAs were identified in GC, and their expressions were positive correlation with some FGF/FGFR. In addition, these FGF/FGFR-related lncRNAs also displayed the same expression trend as some FGF/FGFR between normal and tumor tissues. For instance, both *FOXD2-AS1* and *FGFR4* were upregulated in GC, and their expressions were positively correlative. Previous study indicated that lncRNAs can activate the expression of adjacent genes in a transcript-independent fashion, e.g., enhancer-associated lncRNAs, promotion of genomic domains comprising inter-loci interactions (it could cis-activate the adjacent genes) (Tomita et al., 2015; Statello et al., 2021). The location analysis showed that several FGF/FGFR family members was adjacent to few FGF/FGFR-related lncRNAs, indicating that these lncRNAs may also give play to a similar regulatory effect on the expression of FGF/FGFR. Significantly, more lncRNAs are nonadjacent to FGF or FGFR. We inferred that these lncRNAs may work *via* an indirect regulation in GC, such as the competing endogenous RNA (ceRNA) theory (Tay et al., 2014). Actually, the correlation between FGF/FGFR-related lncRNAs and FGF/FGFR was consistent with the association between lncRNA and mRNA hypothesized by ceRNA theory. Hence, although no direct data supported the regulation of these lncRNAs on FGF/FGFR expression, insight into these FGF/FGFR-related lncRNAs will improve our understanding about FGF/FGFR’s regulatory mechanism in GC, which deserve more experimental works.

Based on FGF/FGFR-related lncRNAs, a four-lncRNA RS model was established in STAD cohort. RS performed a negative correlation with OS and RFS, and could serve as an outstanding prognosis signature in both training and validation cohort. We next analyzed the molecular characteristics in low and high RS groups. Enrichment analysis showed that a great number of oncogenic signatures were enriched in the high RS group, especially KRAS-related gene set. *KRAS* mutation is a frequent oncogenic alteration in a broad range of cancer types, including pancreatic cancer (> 80%), colorectal cancer (> 30%), lung cancer (> 30%), cholangiocarcinoma (> 30%), as well as GC (< 10%) (Polom et al., 2019; Timar and Kashofer, 2020). However, the mutation events of *KRAS* and related pathway were significantly higher in the low RS group than in the high RS group, being inconsistent with the enriched KRAS-related gene set in the latter. Therefore, the expression of wild-type *KRAS* was further analyzed, but no significant difference was observed between the two RS groups. Besides amplification and mutation, KRAS serves as a binary switch in the signal transduction of most growth factor receptors including FGFR, or tyrosine kinase receptor for HGF (MET) (Katoh, 2019; Timar and Kashofer, 2020). Our data indicated that the most of FGF/FGFR family members were significantly up-regulated in the high RS group compared to the low RS group, which may explain the enriched KRAS-related gene sets and other oncogenic signatures in the high RS group. Of note, the expression of the four hub lncRNAs (*DIRC1*, *FGF10-AS1*, *MIR2052HG*, and *POU6F2-AS2*) also displayed a high level in the high RS group. A further investigation about the association between FGF/FGFR and these lncRNAs may throw light on the underlying mechanism of the enriched oncogenic signatures in the high RS tumor.

TME was further estimated. By and large, more immune cells enriched in low RS tumor, while more stromal cells in the high RS tumor. Although more immune checkpoint genes upregulated in the low RS group, it still performed an immune active status compared to the high RS group. In addition, the high RS tumor exhibited potent T-cell exclusion signature. Previous study revealed that stromal cells (fibroblasts and endothelial cells) prevent immune cell infiltrating to TME and inhibit their functions, consequently shaping an immunosuppressive TME (Motz et al., 2014; Denton et al., 2018). The enrichment difference of stromal cells may lead to the different landscape of TME in the low and high RS group. Moreover, TIDE algorithm was used to predict patients’ response to immunotherapy. The low RS group achieved a higher response rate to immunotherapy than the high RS group did. Two points may be responsibility to this prediction. For one thing, the low RS tumor had a high level of PD-L1 and PD-1, which served as the major clinical targets for immunotherapy currently. For another, low RS tumor displayed a high level TMB that can be served as a prediction biomarker for immunotherapy response (Samstein et al., 2019).

Next, the subclass-specific treatment was also put forward. Besides immunotherapy, low RS cohort was also more sensitive to chemotherapy, such as Etoposide and Paclitaxel. Chemotherapy can enhance anti-tumor immune activity *via* several mechanisms (increasing release of cancer antigens, boosting antigen presentation, depletion of immunosuppressive cells, etc.) (Zhu et al., 2021). Accordingly, the combination of chemotherapy is a promising strategy to increase the efficacy of immunotherapy. Clinical trial CHECKMATE-649 and ATTRACTION-4 confirmed the superior efficacy of the combined therapy in treating GC. Accordingly, chemotherapy plus immunotherapy may be suitable for low RS cohort. To identify subclass-specific agents for the high RS cohort, a comprehensive analysis was carried out. Panobinostat and Tivantinib, were identified as most promising drugs for treating high RS cohort. Tivantinib achieves its activity *via* targeting MET, a classical up-stream cascade of KRAS (Timar and Kashofer, 2020), of which related gene set was enriched in high RS group. Panobinostat inhibits specialized enzymes (histone deacetylases, HDACs) that drive oncogenic pathways *via* regulating chromatin remodeling (Ramaiah et al., 2021). Experimental evidences indicated that Tivantinib and Panobinostat exhibited outstanding anti-tumor activity in GC (Kim et al., 2020; Lee et al., 2021). Taken together, Tivantinib and Panobinostat are applicable for high RS tumor with enriched oncogenic signatures. It is noteworthy that Tivantinib as a monotherapy showed a modest efficacy against metastasis GC in a phase II clinical trial (Kang et al., 2014), implying the necessity of precision classification. Further development of our RS model may promote the clinical application of these two drugs in GC. In addition, multi-targeted RTK inhibitor Pazopanib also exhibited superior sensitivity in the high RS tumor, owing to its FGFR1 inhibition activity. These data suggested that FGFR inhibitors may also achieved an encouraging result in the high RS tumor.

Some limitations existed in our study. First, more experimental evidences are necessary for confirming lncRNAs’ regulatory effect on FGF/FGFR. Second, *in vitro* and *in vivo* assays contribute to make clear the efficacy of these candidate agents against GC. In addition, although Panobinostat and Tivantinib have launched for clinical treatment in multiple myeloma and liver cancer, respectively, more clinical evidence are needed to testify our subclass-specific therapy strategy in GC.

In conclusion, some FGF/FGFR-related lncRNAs were identified in GC, and further investigation may improve our understanding about regulatory mechanism of FGF/FGFR. We established a four-lncRNA RS model and clustered GC samples into two subclasses. The two subclasses have distinct molecular characteristics and tumor immunophenotypes. We also put forward subclass-specific therapy for the two cohorts. These results have not only provided new insights into personalized classification model with prognosis signature, but also thrown light on a corresponding precision treatment for tailored risk stratification.

## Data Availability

The datasets presented in this study can be found in online repositories. The names of the repository/repositories and accession number(s) can be found in the article/Supplementary Material.
