# Supplementary material for: FGF/FGFR-related lncRNAs based classification predicts prognosis and guides therapy in gastric cancer
Source: Front Genet. 2022 Aug 29;13:948102. doi: 10.3389/fgene.2022.948102 (PMC9465033; doi:10.3389/fgene.2022.948102)
Supplement: Supplementary file 1 [file DataSheet1.pdf]

Supplementary Material

1 Data Availability Statement

STAD and ACRG cohort were downloaded from TCGA (https://portal.gdc.cancer.gov/) and GEO (https://www.ncbi.nlm.nih.gov/geo) database (Cancer Genome Atlas Research Network, 2014, Cristescu et al., 2015). Annotation file for STAD was from GENCODE database (https://www.gencodegenes.org/; Frankish et al., 2021). The pathology image data was downloaded from TCGA. Drug sensitivity data of CCLs were downloaded from CTRP (https://portals.broadinstitute.org/ctrp) and PRISM (https://depmap.org/portal/prism/; Rees et al., 2016, Corsello et al., 2020). The gene expression data of CCLs were from CCLE project (https://portals.broadinstitute.org/ccle/; Ghandi et al., 2019). CMap analysis was conducted in CMap website (https://clue.io/; Subramanian et al., 2017). Oncogenic signature (c6.all.v7.5.1.symbols) was downloaded from MSigDB (http://www.gsea-msigdb.org/gsea/msigdb; Subramanian et al., 2005). The gene sets of cancer immunity cycle were obtained from TIP website (http://biocc.hrbmu.edu.cn/TIP/; Xu et al., 2018). Immunotherapy response was predicted in TIDE website (http://tide.dfci.harvard.edu/; Jiang et al., 2018). Survival analysis of FGF/FGFR was achieved by Kaplan-Meier Plotter online tool (http://kmplot.com/analysis/index.php?p=service; Szász et al., 2016).

References

Corsello SM, Nagari RT, Spangler RD et al (2020) Discovering the anti-cancer potential of non-oncology drugs by systematic viability profiling. Nat Cancer 1:235-248. doi:10.1038/s43018-019-0018-6

Cristescu R, Lee J, Nebozhyn M et al (2015) Molecular analysis of gastric cancer identifies subtypes associated with distinct clinical outcomes. Nat Med 21:449-456. doi:10.1038/nm.3850

Frankish A, Diekhans M, Jungreis I et al (2021) GENCODE 2021. Nucleic Acids Res 49:D916-d923. doi:10.1093/nar/gkaa1087

Jiang P, Gu S, Pan D et al (2018) Signatures of T cell dysfunction and exclusion predict cancer immunotherapy response. Nat Med 24:1550-1558. https://doi.org/10.1038/s41591-018-0136-1

Network CGAR (2014) Comprehensive molecular characterization of gastric adenocarcinoma. Nature 513:202-209. doi:10.1038/nature13480

Rees MG, Seashore-Ludlow B, Cheah JH et al (2016) Correlating chemical sensitivity and basal gene expression reveals mechanism of action. Nat Chem Biol 12:109-116. doi:10.1038/nchembio.1986

Subramanian A, Tamayo P, Mootha VK et al (2005) Gene set enrichment analysis: a knowledge-based approach for interpreting genome-wide expression profiles. Proc Natl Acad Sci U S A 102:15545-15550. doi:10.1073/pnas.0506580102

Subramanian A, Narayan R, Corsello SM et al (2017) A next generation connectivity map: L1000 platform and the first 1,000,000 profiles. Cell 171:1437-1452.e1417. doi:10.1016/j.cell.2017.10.049

Szász AM, Lánczky A, Nagy Á et al (2016) Cross-validation of survival associated biomarkers in gastric cancer using transcriptomic data of 1,065 patients. Oncotarget 7:49322-49333. doi:10.18632/oncotarget.10337

Xu L, Deng C, Pang B et al (2018) TIP: a web server for resolving tumor immunophenotype profiling. Cancer Res 78:6575-6580. doi:10.1158/0008-5472

2 Supplementary Figures and Tables

2.1 Supplementary Figures

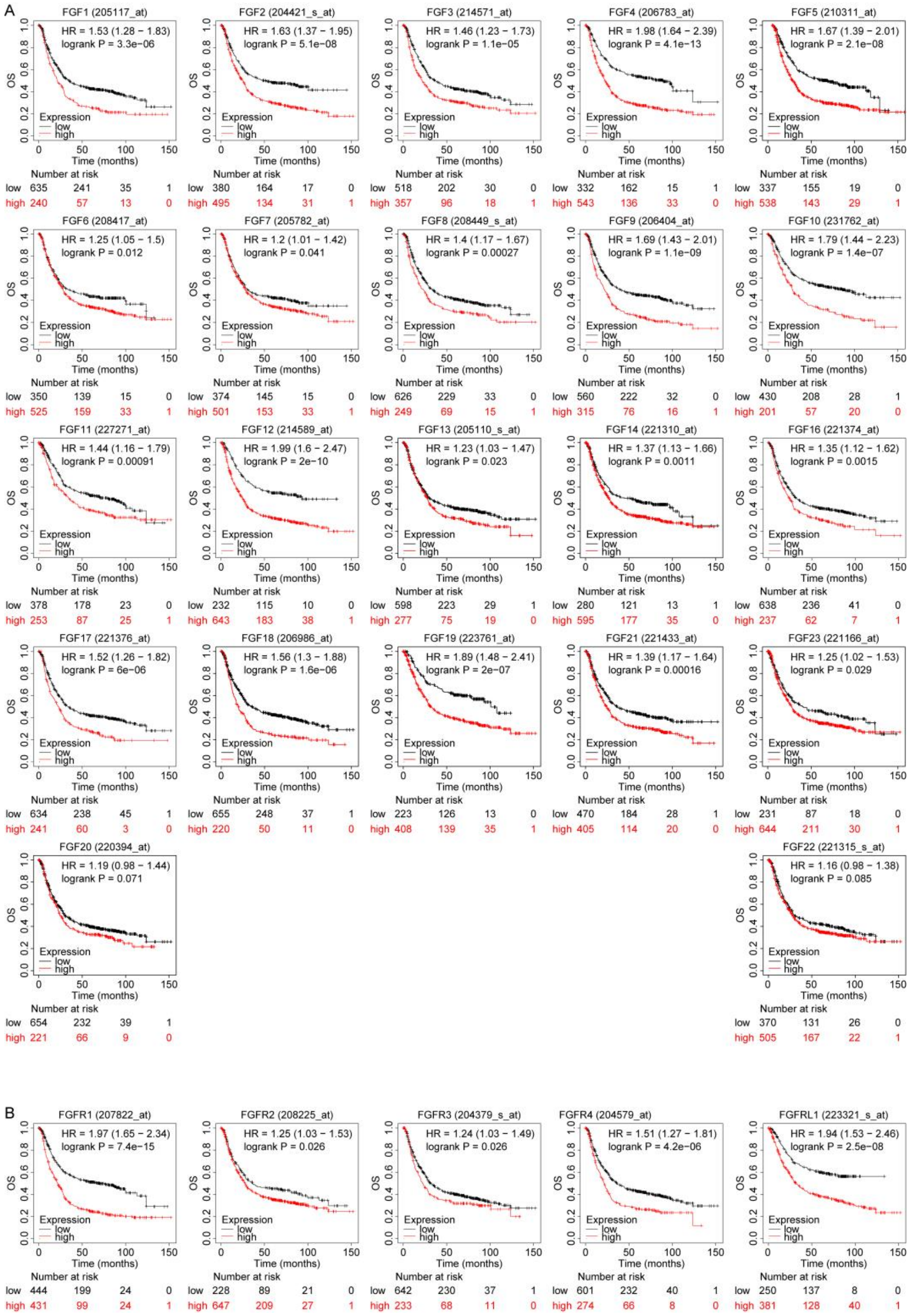

Figure S1. Survival analysis of FGF/FGFR in GC. A & B. Survival analysis of FGFs (A) and FGFRs (B) was performed in GC by using Kaplan-Meier Plotter online tool.

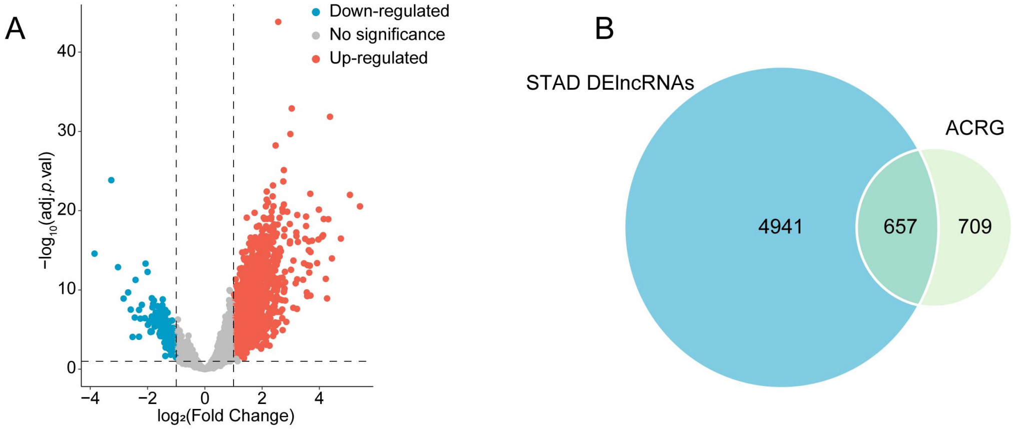

Figure S2. Identification of DElncRNAs. A. Volcano plot showed the DElncRNAs between normal and GC tumor tissues in STAD cohort. B. Venn plot showed 657 lncRNAs shared by STAD and ACRG corhort.

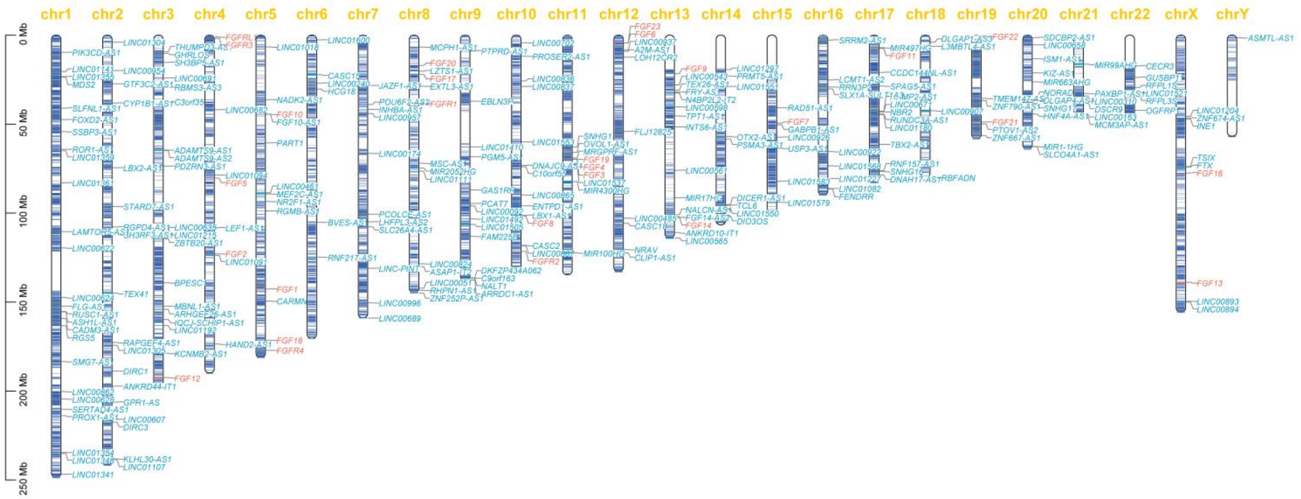

Figure S3. The location of FGF/FGFR family and their related lncRNAs in chromosome.

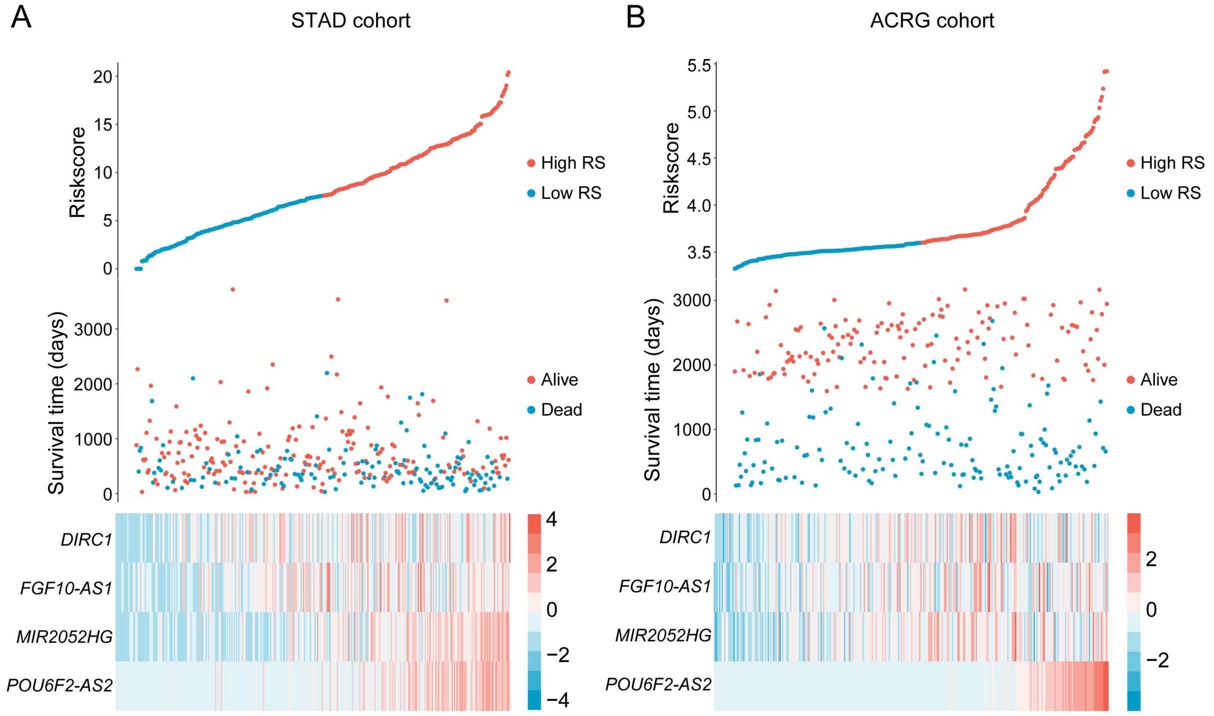

Figure S4. Survival probability was altered with increased RS. A & B. RS was calculated in STAD (A) and ACRG (B) cohorts and displayed as scatter diagram. The expression of the four hub lncRNAs was displayed in heatmap.

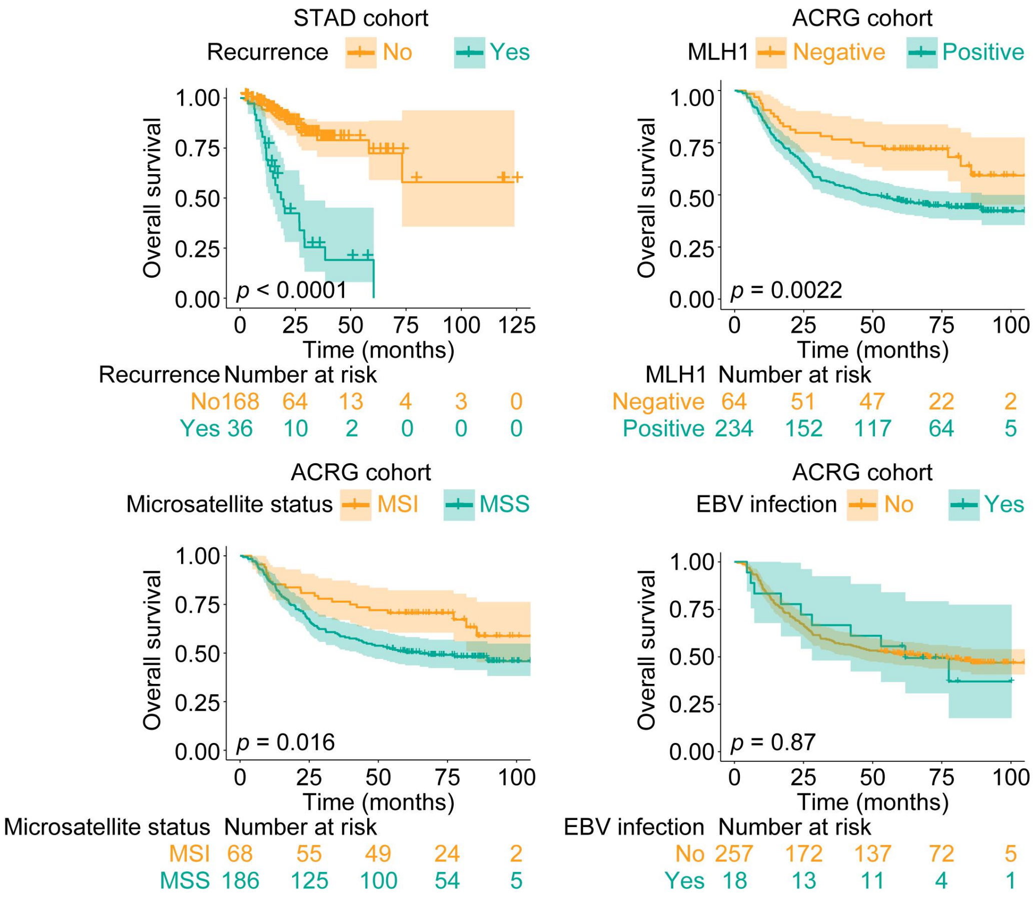

Figure S5. Survival analysis of recurrence, MLH1, microsatellite status and EBV infection in GC.

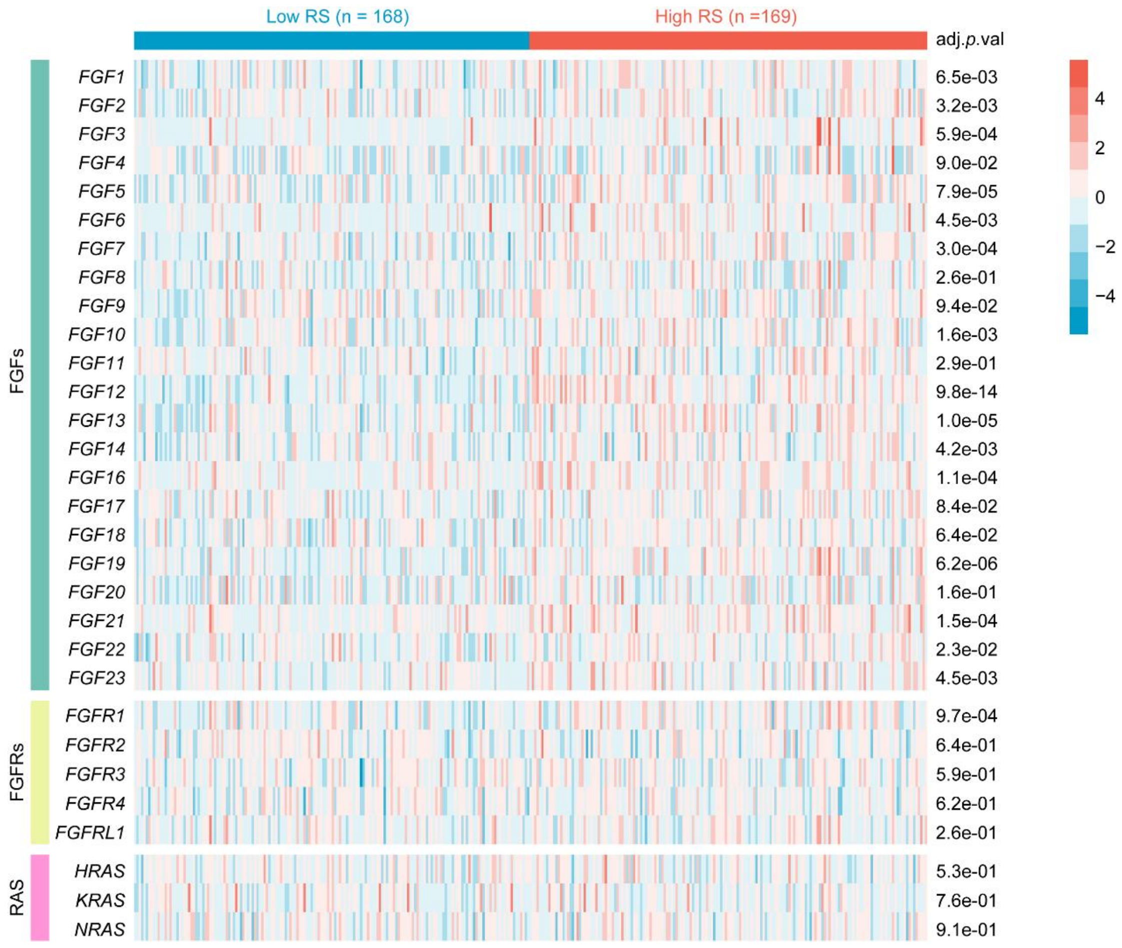

Figure S6. The mRNA expression of FGF/FGFR and RAS between two RS groups.

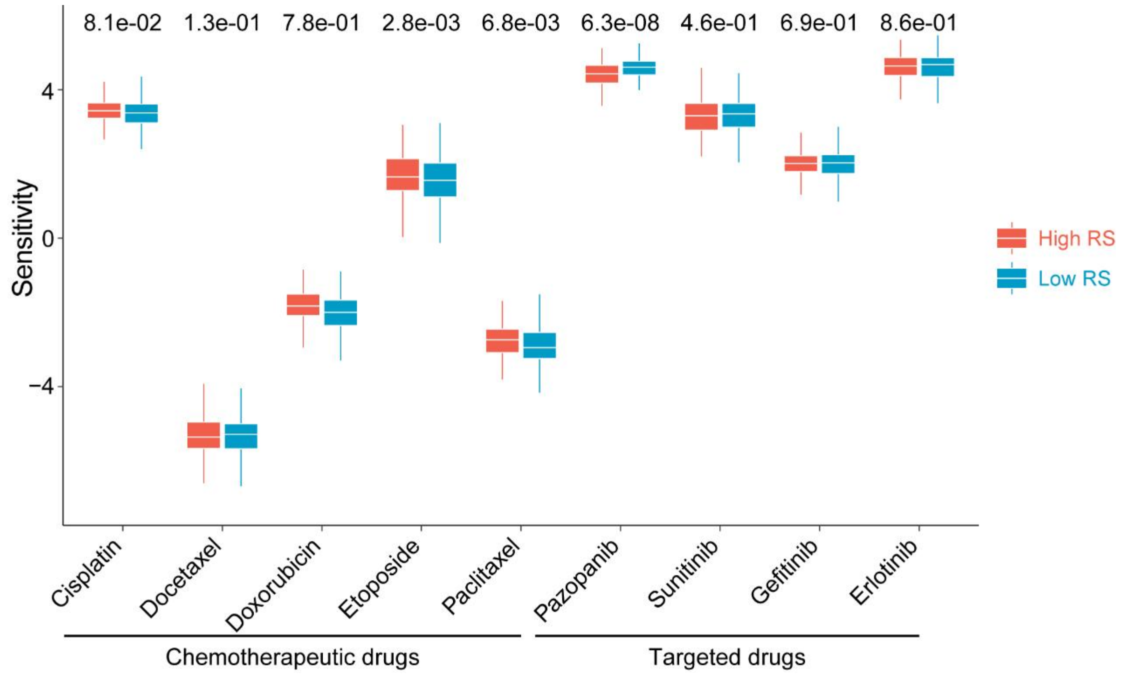

Figure S7. Patients' sensitivity to clinical agents between two RS groups.

2.2 Supplementary Tables

Table S1. The clinical features of patients in STAD and ACRG cohort.

Table S2. Pearson correlation analysis between FGF/FGFR and lncRNAs in STAD.

Table S3. Univariate Cox analysis of lncRNAs in STAD cohort.

Table S4. Multivariate Cox analysis of lncRNAs in STAD cohort.

Table S5. Pearson correlation analysis between RS and differential oncogenic signatures.

Table S6. The results of GSEA.

Table S7. Drugs in CTRP and PRISM database.

Table S8. Human cell lines in CTRP and PRISM database.
